# The Human Milk Oligosaccharide 2′-Fucosyllactose Alleviates Liver Steatosis, ER Stress and Insulin Resistance by Reducing Hepatic Diacylglycerols and Improved Gut Permeability in Obese Ldlr-/-.Leiden Mice

**DOI:** 10.3389/fnut.2022.904740

**Published:** 2022-06-17

**Authors:** Eveline Gart, Kanita Salic, Martine C. Morrison, Martin Giera, Joline Attema, Christa de Ruiter, Martien Caspers, Frank Schuren, Ivana Bobeldijk-Pastorova, Marianne Heer, Yan Qin, Robert Kleemann

**Affiliations:** ^1^Department of Metabolic Health Research, Netherlands Organisation for Applied Scientific Research (TNO), Leiden, Netherlands; ^2^Center for Proteomics and Metabolomics, Leiden University Medical Center, Leiden, Netherlands; ^3^Department of Microbiology and Systems Biology, Netherlands Organisation for Applied Scientific Research (TNO), Zeist, Netherlands; ^4^Human Nutrition, BASF SE, Ludwigshafen, Germany; ^5^Human Nutrition, BASF Pte Ltd., Singapore, Singapore

**Keywords:** human milk oligosaccharides, prebiotics, non-alcoholic fatty liver disease, diacylglycerols, insulin resistance, obesity, gut permeability

## Abstract

Non-alcoholic fatty liver disease (NAFLD) is a complex multifactorial disorder that is associated with gut dysbiosis, enhanced gut permeability, adiposity and insulin resistance. Prebiotics such as human milk oligosaccharide 2′-fucosyllactose are thought to primarily improve gut health and it is uncertain whether they would affect more distant organs. This study investigates whether 2′-fucosyllactose can alleviate NAFLD development in manifest obesity. Obese hyperinsulinemic Ldlr-/-.Leiden mice, after an 8 week run-in on a high-fat diet (HFD), were treated with 2′-fucosyllactose by oral gavage until week 28 and compared to HFD-vehicle controls. 2′-fucosyllactose did not affect food intake, body weight, total fat mass or plasma lipids. 2′-fucosyllactose altered the fecal microbiota composition which was paralleled by a suppression of HFD-induced gut permeability at *t* = 12 weeks. 2′-fucosyllactose significantly attenuated the development of NAFLD by reducing microvesicular steatosis. These hepatoprotective effects were supported by upstream regulator analyses showing that 2′-fucosyllactose activated ACOX1 (involved in lipid catabolism), while deactivating SREBF1 (involved in lipogenesis). Furthermore, 2′-fucosyllactose suppressed ATF4, ATF6, ERN1, and NUPR1 all of which participate in endoplasmic reticulum stress. 2′-fucosyllactose reduced fasting insulin concentrations and HOMA-IR, which was corroborated by decreased intrahepatic diacylglycerols. In conclusion, long-term supplementation with 2′-fucosyllactose can counteract the detrimental effects of HFD on gut dysbiosis and gut permeability and attenuates the development of liver steatosis. The observed reduction in intrahepatic diacylglycerols provides a mechanistic rationale for the improvement of hyperinsulinemia and supports the use of 2′-fucosyllactose to correct dysmetabolism and insulin resistance.

## Graphical Abstract

Created with BioRender.com. 
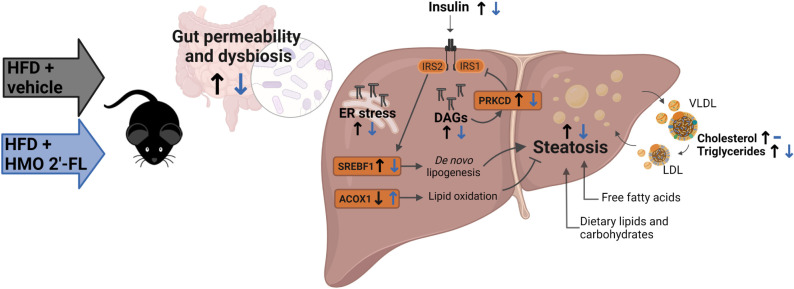

## Introduction

Increased energy intake, particularly from diets rich in sucrose and saturated fat, leads to the development of obesity which is frequently associated with intrahepatic lipid accumulation (steatosis), an early hallmark of non-alcoholic fatty liver disease (NAFLD) (1). NAFLD is a complex multifactorial disorder that is linked to microbial dysbiosis, gut permeability, and insulin resistance (IR) (1), all of which are considered to be drivers of disease progression. In the liver, triacylglycerols (TAGs) start to accumulate when the rates of hepatic lipid uptake and production exceed the rates of lipid oxidation and export (*via* VLDL particles). Insulin resistance is accompanied by hyperinsulinemia, which can drive hepatic lipid accumulation by stimulating *de novo* lipogenesis *via* the transcriptional master regulator SREBP1c (2). At the same time, specific lipids may build up in the liver, which can exacerbate hepatic IR thereby creating a vicious circle (3). Critical molecules that directly participate in the development of insulin resistance at tissue level are lipids such as diacylglycerols (DAGs), which are intermediate products of triacylglycerol (TAG) synthesis (3). In contrast to TAGs—which are considered to be a safe storage form of lipids—DAGs are bioactive lipids that have been shown to directly interfere in insulin signaling. Conversely, treatments that lower DAGs can improve IR (3).

Prebiotics are non-digestible food ingredients such as complex oligosaccharides (4). It is thought that these non-digestible oligosaccharides can provide a health benefit to the host by promoting the growth or activity of specific microorganisms in the gut, for instance by stimulating the growth of Bifidobacterium genera (5). A recent meta-analysis showed that treatment with prebiotics can improve liver integrity markers such as ALT in NAFLD patients, suggesting an effect of prebiotics beyond the gut on the liver, which plays an essential role in whole-body metabolism (6). 2′-fucosyllactose (2′-FL) is the most prevalent human milk oligosaccharide (HMO) present in human breast milk, and has been reported to protect against pathogens (7). Furthermore, it can stimulate the growth of beneficial gut bacteria (8) and attenuate ethanol-induced liver damage in a rodent model (9). Putative effects of the HMO 2′-FL on NAFLD have not been studied so far, and it is unknown whether 2′-FL can reduce NAFLD pathology development in a therapeutic setting, i.e., when obesity, dyslipidemia (high VLDL/LDL) and hyperinsulinemia are already manifest at the start of 2′-FL supplementation.

To study the putative hepatoprotective effects of the HMO 2′-FL under conditions of hyperinsulinemia, dyslipidemia and obesity we used Ldlr-/-.Leiden mice, a high fat diet (HFD)-inducible model of NAFLD/NASH that recapitulates phenotypical and molecular disease profiles of NAFLD patients (10–12). After a run-in of 8 weeks on HFD, obese hyperinsulinemic mice received 2′-FL by oral gavage and were compared to HFD-control mice receiving saline. After a total treatment period of 20 weeks, liver histology was studied in conjunction with lipidomics and transcriptomics to gain insight into the metabolic processes that are affected in the liver.

## Materials and Methods

### Animal Experiment

Male Ldlr-/-.Leiden mice were bred and housed in the American Association for Accreditation of Laboratory Animal Care (AAALAC)-accredited animal facility at TNO (Leiden, Netherlands). They were group-housed (four to five mice per cage) in Macrolon type 2L cages in a clean-conventional animal room (relative humidity 55 ± 10%, temperature 20–24°C, light cycle 07:00 to 19:00) with *ad libitum* access to food and water. To induce obesity, mice were fed a HFD (D12451, Research Diets Inc., New Brunswick, United States; containing 20 kcal% protein, 35 kcal% carbohydrate, 45 kcal% fat mainly from lard) as reported previously (13) and mice on standard rodent chow (Sniff-R/M-V1530, Uden, Netherlands) were used as a reference.

**FIGURE 1:**
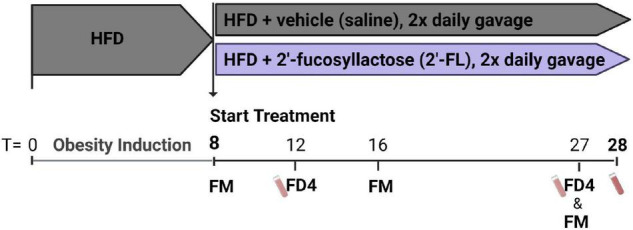
Ldlr–/–.Leiden mice were pretreated with a HFD for 8 weeks to induce an obese phenotype with hyperinsulinemia. These mice were then divided into two groups. One group of mice was treated with 2′-fucosyllactose (2′-FL) by oral gavage twice daily (750 mg/kg/day) until week 28, and subsequently compared with HFD + vehicle control mice receiving twice daily saline (*n* = 18/group). At week 8, 16 and 27 feces were collected for 16S microbiota profiling (fecal microbiota; FM). Blood samples were taken at week 12 and 27 for gut permeability measurements (FD4) and in week 28 sacrifice plasma was collected.

After a run-in of 8 weeks on HFD, obese hyperinsulinemic mice were matched into 2 groups based on body weight, blood glucose, plasma cholesterol and triglycerides concentrations. One group of mice was treated with 2′-fucosyllactose (2′-FL) by oral gavage twice daily (750 mg/kg/day) until week 28, and compared with the HFD + vehicle controls that received oral saline twice daily (*n* = 18/group). An experimental outline is provided in Figure 1. Blood samples, gut permeability (by FD4 tests) and body composition (by EchoMRI) were longitudinally analyzed during the study. Fecal samples were collected from individual mice for 16S microbiota profiling. Mice were terminated in week 28 by gradual-fill CO_2_ asphyxiation. At sacrifice, livers and other organs were isolated and immediately snap-frozen in liquid N_2_ and/or fixed in formalin as previously described (14).

### Gut Permeability and Microbiota Composition Analysis

An *in vivo* FD4 assay was used to measure functional gut permeability as described previously (15). In short, plasma concentrations of FITC-labeled dextran (three to five kDa FD4; Sigma, St. Louis, MO, United States) were determined 4 h after FD4 administration *via* oral gavage, indicative of paracellular permeability of the intestinal barrier.

Feces were collected from individual mice in week 8 (pre-treatment), as well as at *t* = 16 weeks and *t* = 27 weeks. These fecal samples were mechanically homogenized and genomic DNA was isolated using the AGOWA Mag mini kit (DNA Isolation Kit, AGOWA, Berlin, Germany) according to the manufacturer’s instructions. Microbiota composition was determined using an established PCR-based amplification method of the hypervariable region V4 a fragment of 16S rRNA as described previously (14). Subsequent bioinformatical analyses of the dynamical changes in microbiota composition and the microorganisms (on genera level) were analyzed over time as detailed in Gart et al. (14).

### Blood Chemistry

Fasting (5 h) blood samples were taken from the tail vein to prepare EDTA plasma. Blood glucose was determined during blood sampling using a hand-held glucometer (Freestyle Freedom Light, Abbott Laboratories, Lake Bluff, IL, United States). Plasma insulin, leptin, adiponectin, cholesterol, triacylglycerols, ALT, CK-18M30 were determined as described (16). ELISA assays were used to measure plasma serum amyloid A (SAA; KMA0021 Thermo Fisher Scientific, Waltman, United States), MCP-1/CCL2 (DY479 R&D, Minneapolis, United States), MIF (DY1978 R&D, Fetuin A (DY1563 R&D) and LPS binding protein (LBP; HK205-02 Hycult Biotech, Uden, Netherlands) according to manufacturer’s protocol.

Lipoprotein profiles were analyzed by first separating lipoproteins into fractions using fast protein liquid chromatography (FPLC) with an AKTA apparatus (Pharmacia, Roosendaal, Netherlands), as previously described (17, 18). Subsequently, total cholesterol and triacylglycerols were measured in the fractions collected for profiling with enzymatic assays (Roche diagnostics, Basel, CHF) (19–21).

### Liver Biochemistry and Histology

Liver biopsies (2.5 mg) were homogenized and processed to extract lipids. Lipids were quantified with the Lipidyzer platform as previously described (22, 23). Lipids were grouped into classes (triacylglycerols (TAGs), diacylglycerols (DAGs), monoacylglycerols, free fatty acids, cholesteryl esters, phosphatidylcholines, phosphatidylethanolamines, sphingolipids, ceramides etc.). The concentrations of the lipids were expressed as nmol/mg tissue.

Histopathological analyses of liver steatosis, hepatocellular hypertrophy and inflammation were performed on 3-μm-thick hematoxylin-eosin-stained cross sections of the medial lobe using a standardized method for rodent liver histopathology based on the human NAS scoring system (24). The cross-sectional liver area was assessed by a board-certified pathologist to determine the percentage area with microvesicular steatosis and macrovesicular steatosis as well as the area with abnormally enlarged cells (hypertrophy) essentially as detailed in Gart et al. (25). Lobular inflammation was assessed by counting the number of inflammatory aggregates which were expressed per mm^2^ as reported (25).

### Gene Expression Analysis Using Next-Generation Sequencing and Subsequent Pathway Analysis

RNA was isolated from livers (*n* = 18 HFD + vehicle and *n* = 18 HFD + 2′-FL) using RNA-Bee (Bio-Connect, Huissen, Netherlands) and purified using PureLink RNA Mini Kit (Thermo Fisher Scientific). RNA concentration was determined and RNA quality was assessed as reported in Gart et al. (16). Strand-specific messenger RNA sequencing libraries for the Illumina (llumina NovaSeq6000, San Diego, CA, United States) platform were generated Paired-End 150 bp for approximately 20 million Paired-End reads per sample at Genomescan (Leiden, Netherlands). The sequences were filtered, trimmed and subjected to a QC procedure as described previously (26). These files were then merged and aligned to the reference genome “Mus_musculus.GRCm38.gencode.vM19”. To count the read mapping frequency per gene Htseq-count 0.6.1p1 was used, and resulting count files (data publicly available via the NCBI Gene Expression Omnibus (GEO) database https://www.ncbi.nlm.nih.gov/geo/ under accession number GSE195862) served as input for the differentially expressed genes (DEGs) analysis using the DESeq2-method (27). DEGs were used as an input for upstream regulator and pathway analysis through Ingenuity Pathway Analysis (IPA) (28). IPA uses gene expression data of all known downstream target genes to predict activation or deactivation of an upstream regulator or pathway as reported (25). Positive Z-scores (Z ≥ 2) indicate an enhanced activity of an upstream regulator while negative Z-scores (Z ≤ −2) indicate a reduced activity of an upstream regulator (25), while the *P*-value indicates significant enrichment of the target genes downstream of a regulator.

### Statistics

The present study tested the null hypothesis that 2′-FL does not improve disease parameters relative to saline controls (HFD + vehicle). All statistical analyses were performed using SPSS Version 25 (SPSS Inc., Chicago, IL, United States). Normal distribution of variables was analyzed with the Shapiro–Wilk test, assuming normality at *p* > 0.05. For normally distributed variables, and independent sample *t*-test was used (1-sided). In case the data was not normally distributed, a Mann–Whitney test was used (1-sided). Gut permeability data from multiple time points (*t* = 12 and *t* = 27) was analyzed with a 2-way ANOVA. Correlation analyses were performed by Spearman’s rank correlation analysis. *P*-values < 0.05 were considered statistically significant. Data are represented as means ± SD. IPA analysis to determine differentially expressed genes were based on Fisher’s exact test (α = 0.01).

## Results

### HMO 2′-FL Does Not Affect Adiposity or Metabolic Parameters

**FIGURE 2:**
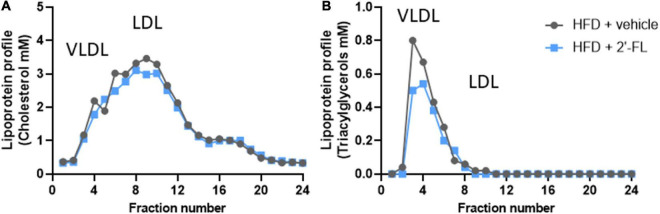
Lipoprotein profiles were analyzed in fasting plasma (pooled from *n* = 18 mice per treatment group) by fast protein liquid chromatography (FPLC). The plasma is fractionated and lipoprotein particles are collected in the respective fractions. In these fractions, **(A)** cholesterol and **(B)** triglyceride concentrations were analyzed and plotted as profiles, which demonstrate changes in lipoprotein particles to which the plasma lipids are associated with.

Ldlr-/-.Leiden mice with a starting body weight of 27.0 ± 2.0 gram were fed an energy-dense HFD during an 8-week run-in period to induce obesity (41.5 ± 4.2 grams). The weight gain on HFD was found to be about threefold higher than on a chow diet (chow mice weigh on average 31.6 ± 3.2 grams). After 8 weeks the intervention was started and mice received either 2′-FL or vehicle control until week 28. During the entire study, the average food intake was comparable between the two groups (Table 1). At *t* = 28 weeks, both groups gained a comparable amount of body weight, fat mass and lean mass. Visceral adiposity, reflected by the mass of epididymal (eWAT) and mesenteric (mWAT) adipose tissue depots, was also comparable between the groups, as was the weight of the subcutaneous depot (sWAT). Consistent with this, the plasma concentrations of the adipokines leptin and adiponectin were consistent with an obese phenotype and were not affected by 2′-FL compared to the HFD + vehicle controls. Markers associated with inflammation including plasma MCP-1/CCL2 was significantly decreased with 2′-FL, while plasma MIF, SAA and Fetuin A were comparable in 2′-FL and HFD + vehicle treated controls. Plasma total cholesterol and triacylglycerol (TAG) levels were comparable between the groups. Lipoprotein profile analysis showed that cholesterol confined to apoB-containing (V)LDL-sized particles was comparable, while TAGs confined to (V)LDL-sized particles were somewhat reduced in the 2′-FL group (Figure 2).

**TABLE 1:** Body composition, energy intake and metabolic and inflammatory parameters after 28 weeks.

	HFD + vehicle	HFD + 2′-FL
Average food intake (g/day)	2.5 ± 0.2	2.6 ± 0.3
Body weight (g)	46.8 ± 4.2	47.4 ± 4.7
Fat mass (g)	19.5 ± 3.1	19.9 ± 3.6
Fat mass (%)	42.5 ± 4.2	42.5 ± 4.0
Lean mass (g)	26.2 ± 2.1	26.6 ± 3.6
eWAT (g)	2.2 ± 0.4	2.4 ± 0.6
mWAT (g)	1.0 ± 0.3	0.9 ± 0.3
sWAT (g)	2.6 ± 0.7	2.5 ± 0.6
Plasma leptin (ng/ml)	48.4 ± 13.5	46.4 ± 10.5
Plasma adiponectin (μg/ml)	9.9 ± 3.5	10.0 ± 2.8
Plasma MCP-1/CCL2 (pg/ml)	15.7 ± 8.7	9.6 ± 4.0*
Plasma MIF (μg/ml)	32.0 ± 14.5	22.1 ± 14.9
Plasma SAA (μg/ml)	18.8 ± 4.6	19.5 ± 4.1
Plasma Fetuin A (μg/ml)	46.1 ± 7.7	44.4 ± 10.8
Plasma cholesterol (mM)	31.0 ± 7.8	29.9 ± 8.4
Plasma triacylglycerol (mM)	6.5 ± 2.4	5.8 ± 2.6

eWAT = epididymal white adipose tissue, mWAT = mesenteric white adipose tissue and sWAT = subcutaneous white adipose tissue. Data are presented as mean ± SD. Asterisks indicate a significant difference relative to HFD + vehicle control (*p < 0.05).

### 2′-FL Supplementation Reduces Liver Steatosis

**FIGURE 3:**
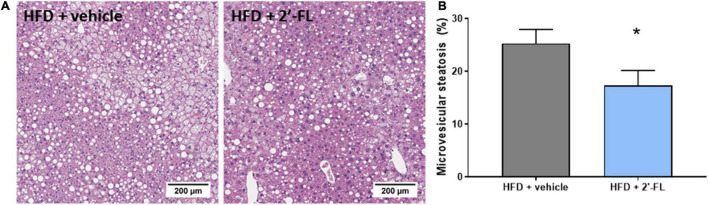
Human milk oligosaccharide 2′-FL reduced the accumulation of lipids in small lipid droplets referred to as microvesicular steatosis. **(A)** Representative images **(B)** percentage of hepatic cross-section affected with microvesicular steatosis. Data is from *n* = 18 mice per treatment group shown as mean ± SEM, *p < 0.05.

Plasma concentrations of ALT, a liver integrity marker associated with NAFLD development, tended to be decreased with 2′-FL (*p* = 0.07), while CK18-M30 and total liver weight were not altered compared to the HFD + vehicle controls (Table 2). Subsequent histological analysis of NAFLD in liver cross-sections demonstrated that 2′-FL reduced the total steatotic area with borderline significance (−19%; *p* = 0.051). This prompted us to perform a more refined analysis of steatosis subtypes, i.e., macrovesicular and microvesicular steatosis, the former being associated with lobular inflammation (29), the latter being associated with mitochondrial dysfunction and oxidative stress (30). Remarkably, 2′-FL did not affect macrovesicular steatosis, and its anti-steatotic effect was almost fully attributable to a suppression of microvesicular steatosis (−32%; *P* < 0.05) (Figure 3). This hepatoprotective effect was further supported by an effect on hepatocellular hypertrophy, i.e., the liver area covered with abnormally enlarged cells (−32%; *P* < 0.05) (Table 2). The number of inflammatory aggregates reflecting lobular inflammation was unaffected (Table 2).

**TABLE 2:** Liver integrity markers, liver weight and NAFLD score after 28 weeks.

	HFD + vehicle	HFD + 2′-FL
Plasma ALT (U/L)	210.8 ± 95.3	163.7 ± 85.9
Plasma CK18-M30 (U/mL)	305.6 ± 67.1	334.7 ± 82.4
Liver weight (g)	2.6 ± 0.7	2.5 ± 0.6
Total steatosis (%)	47.4 ± 16.2	38.1 ± 17.3^#^
Macrovesicular steatosis (%)	22.1 ± 7.5	20.8 ± 8.1
Hepatocellular hypertrophy (%)	30.6 ± 15.4	21.1 ± 14.0*
Inflammatory aggregates (per mm2)	6.8 ± 16.9	3.9 ± 9.7

Asterisks indicate a significant difference relative to HFD + vehicle control (*p < 0.05, ^#^p = 0.051). Data shown as mean ± SD.

### 2*′*-FL Modulates Upstream Regulators Involved in Lipid Metabolism and ER Stress

To gain more insight into the effects of 2′-FL on metabolic processes in the liver, a genome-wide gene expression analysis was performed using NGS and datasets were analyzed with Ingenuity Pathway Analysis (IPA). IPA predicts the activation state of critical upstream regulators (e.g., signaling molecules, transcription factors) based on the expression pattern of their downstream genes. Consistent with the development of steatosis in the HFD group, important upstream regulators involved in lipid catabolism (peroxisomal acyl-CoA oxidase 1 (ACOX1), AMP kinase (AMPK) and mitochondrial biogenesis (PPARGC1a) were all inactivated with HFD (Table 3). 2′-FL counteracted this HFD effect and specifically reactivated ACOX1 indicating that 2′-FL supplementation enhanced beta-oxidation. Furthermore, the activity of sterol regulatory element-binding transcription factor 1 (SREBF1), a positive regulator of fatty acid synthesis genes, was suppressed by 2′-FL which is in line with reduced plasma insulin levels and the observed lowering of TAG in (V)LDL particles. In addition, HFD elevated the activity of protein kinase C delta (PRKCD) which impairs insulin signaling, and this activation was counter regulated with 2′-FL. Since PRKCD can be activated by DAGs, a decreased activity also suggests a lower amount of intrahepatic DAGs with 2′-FL. Several regulators associated with endoplasmic reticulum (ER) stress, e.g., ATF4, ATF6, ERN1, NUPR1, were activated with HFD indicating increased ER stress in the control group. 2′-FL significantly attenuated the effects of HFD, indicating an overall suppression of the ER stress response. Altogether, the NGS analyses support an improvement of hepatic lipid handling by 2′-FL that is accompanied by beneficial effects on the ER stress response. Consistent with these upstream regulator effects, the canonical pathway “Nrf2-mediated oxidative stress response” was attenuated with 2′-FL (Z = −1.6, *p* < 0.05) (Supplementary Table 1).

**TABLE 3:** Upstream regulator analysis of factors controlling metabolic homeostasis in the liver.

	HFD + vehicle vs Chow		HFD + 2′-FL vs HFD + vehicle	
	
	Z-score	*p*-value	Z-score	*p*-value
ACOX1	−5.8	0.000	2.2	0.037
AMPK	−2.2	0.000	N/A	1.000
ATF4	1.9	0.000	−2.6	0.000
ATF6	0.5	0.007	−1.9	0.000
ERN1	0.9	0.037	−2.4	0.009
NRF1	−0.9	0.000	0.6	0.000
NUPR1	2.8	0.000	−1.2	0.005
PPARGC1A	−4.2	0.000	0.1	0.005
PRKCD	3.8	0.000	−2.4	0.011
SREBF1	0.2	0.000	−1.7	0.005

*The activity of an upstream regulator was predicted based on gene expression changes of all downstream target genes. A negative Z-score indicates inhibition of the respective regulator or pathway (green color) and a positive Z-score indicates activation (red color). The p-value < 0.05 in gray indicates significant enrichment of the target genes downstream of a regulator, i.e., that more downstream genes are affected than can be expected by chance. N/A indicates an insufficient number of differentially expressed genes to predict the activation state of an upstream regulator.*

We next investigated effects exerted by 2′-FL supplementation during the experiment that may explain the reduced development of liver steatosis, with particular emphasis on gut permeability and insulin resistance.

### 2′-FL Modulates Microbiota and Improves Gut Permeability

**FIGURE 4:**
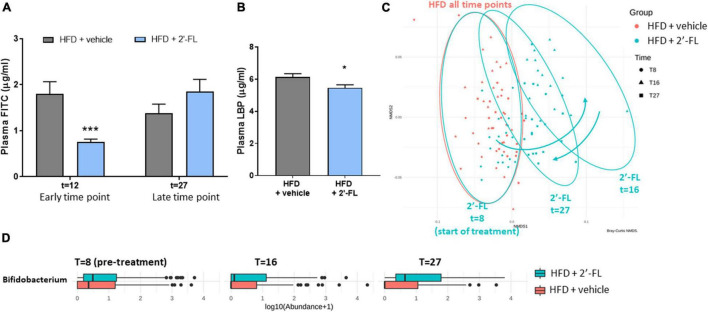
Human milk oligosaccharide 2′-FL improves gut permeability during early NAFLD development. **(A)** Gut permeability measured by FITC-labeled dextran at an early (at *t* = 12 w) and late (at *t* = 27 w) time point of the disease process equaling 4 and 19 weeks of 2′-FL supplementation, respectively. **(B)** Plasma LPS-binding protein (LBP) measured in week 28 of the study. Data is from *n* = 18 mice per treatment group presented as mean ± SEM, *p < 0.05, ***p < 0.001 compared to HFD + vehicle. **(C)** Fecal microbiota composition was analyzed by 16S sequencing and composition changes over time were visualized by non-metric multidimensional scaling (NMDS) using the Bray-Curtis index. Every dot represents the microbiota composition of one mouse. A greater distance between dots indicates more dissimilarity. Blue arrows indicate 2′-FL induced microbiota composition shifts. **(D)** Fecal Bifidobacterium abundance over time.

Orally administered FITC-labeled dextran (FD4) was used to measure the functional integrity of the gut. The amount of FD4 retrieved in plasma in Ldlr-/-.Leiden mice on chow was 1.2 ± 0.5 μg/mL (not shown). At *t* = 12 weeks, HFD feeding resulted in an increased gut permeability and this HFD effect was strongly attenuated with 2′-FL (−55%; *p* < 0.05), i.e., after only 4 weeks of 2′-FL supplementation (Figure 4A). The effect of 2′-FL on gut permeability was not observed anymore at week 27 with the FD4 test, while plasma LBP was significantly decreased with 2′-FL after 28 weeks (Figure 4B).

To investigate whether the reduced gut permeability during the development of NAFLD was associated with specific changes in microbiota composition, 16S sequencing was performed on feces collected prior to the intervention (at *t* = 8 weeks) and at time points close to the FD4 tests. Permutation tests enabled the statistical testing of differences in entire microbiota composition between experimental groups, and showed that, prior to the 2′-FL intervention, the HFD + vehicle and HFD + 2′-FL groups were comparable as expected. The similarity of their microbiota composition is visualized in an NMDS plot (Figure 4C) showing a great overlap between the groups at *t* = 8 weeks. While the microbiota of the HFD + vehicle remained stable during the study, 2′-FL significantly altered the microbiota composition as revealed by a marked shift to the right at *t* = 16 weeks, indicating a higher dissimilarity relative to respective controls early during the intervention. Toward the end of the study, at *t* = 27 weeks, the microbiota composition was still significantly different from the HFD-controls, however, the dissimilarity was smaller as the dots shifted back in the direction of the HFD controls. In addition to these shifts on the level of the entire microbiome, analysis of specific genera over time demonstrated that 2′-FL gradually increased the Bifidobacterium content over time (Figure 4D). Altogether, 2′-FL transiently reduced gut permeability and led to pronounced changes of fecal microbiota composition during the time in which NAFLD developed.

### 2*′*-FL Lowers Hyperinsulinemia and HOMA-IR and Reduces Liver Diacylglycerols

In the fasting state, blood glucose is mainly (∼90%) derived from the liver (31). Fasting blood glucose at the end of the treatment period was not affected by 2′-FL (Table 4). By contrast, fasting plasma insulin concentrations, which increased strongly in response HFD feeding (from 1.6 ± 0.6 ng/mL at the start of the experiment to 12.5 ± 5.1 ng/mL), were significantly lowered by 2′-FL. Also, insulin resistance assessed by HOMA-IR was significantly reduced in the 2′-FL group, suggesting improved insulin action in livers of 2′-FL fed mice.

**TABLE 4:** Glucose, insulin and insulin resistance (HOMA-IR) after 28 weeks.

	HFD + vehicle	HFD + 2′-FL
Blood glucose (mmol/l)	7.9 ± 0.8	8.1 ± 1.2
Plasma insulin (ng/ml)	12.5 ± 5.1	9.1 ± 4.0*
HOMA-IR	110.2 ± 49.8	83.7 ± 41.5*

Asterisk indicates a significant difference relative to the HFD + vehicle control group (*p < 0.05). Data shown as mean ± SD.

**FIGURE 5:**
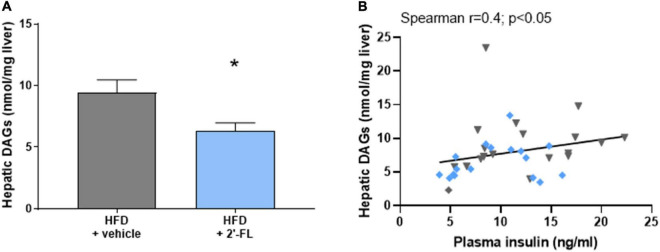
Human milk oligosaccharide 2′-FL reduced **(A)** total intrahepatic diacylglycerols (DAGs). **(B)** Total DAGs correlated with fasting plasma insulin concentrations. Data is from *n* = 18 mice per treatment group shown as mean ± SEM, *p < 0.05.

We therefore analyzed the concentrations of diacylglycerols (DAGs) in the liver as this lipid species blocks insulin action at the level of its receptor, an effect that can be dissociated from inflammation-mediated IR (3). In response to HFD feeding, liver DAGs in the HFD + vehicle-treated group were 9.4 ± 4.4 nmol/mg liver and thus markedly elevated compared to littermates on chow (2.6 ± 0.8 nmol/mg liver; not shown). The HFD-induced accumulation of intrahepatic DAGs was significantly suppressed by 2′-FL, which is consistent with a reduction of HOMA-IR by 2′-FL relative to vehicle control (Figure 5A). The relationship between fasting plasma insulin concentrations and intrahepatic DAGs was further supported by a significant correlation between the two parameters (Figure 5B). Altogether, 2′-FL attenuated gut permeability and IR thereby providing a rationale for the reduction of NAFLD development.

## Discussion

In the current study we investigated the potential hepatoprotective effects of the human milk oligosaccharide 2′-fucosyllactose (2′-FL) in a therapeutic setting, i.e., we started its supplementation when obesity, dyslipidemia and hyperinsulinemia were already manifest. Long-term supplementation (20 weeks) with 2′-FL counteracted the detrimental effects of HFD feeding on dysbiosis and gut permeability, which was accompanied by an increase in the abundance of Bifidobacterium. In the liver, 2′-FL specifically suppressed the accumulation of lipids in small intracellular droplets, so-called microvesicular steatosis. These hepatoprotective effects were supported by functional transcriptome analysis, which demonstrated improved lipid handling (by activating the peroxisomal enzyme ACOX1 and deactivating the transcription factor SREBF1). Improved hepatic lipid handling by 2′-FL was accompanied by beneficial effects on the ER/cellular stress response because 2′-FL counter-regulated HFD-dependent induction of ATF4, ATF6, ERN1, NUPR1 and the “Nrf2-mediated oxidative stress response” pathway. Moreover, 2′-FL significantly reduced fasting insulin concentrations and HOMA-IR, an observation that is mechanistically supported by a significant decrease in intrahepatic bioactive DAGs and deactivation of PKC-delta which has been shown to block insulin signaling upon binding to DAGs (3, 32).

The current study is the first to investigate the effect of long-term supplementation with 2′-FL on NAFLD development and demonstrates hepatoprotective effects independent of changes on body weight, food intake or plasma lipids. The absence of an effect on body weight and food intake is in line with short-term studies (6–8 weeks) that used comparably low doses of 2′-FL (1 and 2% w/v) as herein (2.5% w/w) (33). In these short-term studies only the higher doses of 2′-FL (5 and 10% w/v) decreased body weight, which was also associated with a lowering of caloric intake (33, 34). In line with our findings, no major effect on plasma lipids was observed in a meta-analysis of other types of prebiotic treatments in ultrasound- or biopsy-confirmed NAFLD patients (6).

The supplementation of 2′-FL in diet-induced obese and hyperinsulinemic mice resulted in significantly lower microvesicular steatosis. In general, hepatic steatosis occurs when hepatic uptake and production of lipids is higher than the combined lipid export *via* VLDL and lipid oxidation. As TAGs confined to VLDL were slightly reduced in this study, it appears that 2′-FL did apparently *not* stimulate hepatic lipid export to reduce steatosis. Our observation rather suggests that 2′-FL may improve lipid utilization by increasing fatty acid oxidation and/or decreasing *de novo* lipogenesis. Indeed functional transcriptomic analysis demonstrated that 2′-FL specifically activated ACOX1, the rate-limiting enzyme in peroxisomal beta oxidation (35, 36) with an important role in lipid catabolism. At the same time 2′-FL deactivated SREBF1, the transcriptional master regulator of *de novo* lipogenesis (2), which is itself positively regulated by insulin. Fasting insulin levels were strongly increased with HFD and attenuated by 2′-FL, which is consistent with the observed activation/deactivation of SREBF1. This is particularly relevant for the situation in NAFLD patients, in which it has also been demonstrated that hyperinsulinemia as a consequence of IR is a key driver of *de novo* lipogenesis (37). Moreover, insulin also plays an important role in regulation of fatty acid fluxes to the liver because it inhibits TAG lipolysis in adipose tissue (38). Under conditions of peripheral IR however, the fluxes of fatty acids from extrahepatic organs to the liver can increase markedly because insulin is no longer able to put the brakes on lipolysis (3). These peripheral effects in the adipose organ, together with an excessive dietary intake of carbohydrates and lipids further promotes the development of liver steatosis. The observed reductions in fasting insulin and HOMA-IR with 2′-FL are in line with results from a human study, in which increased serum concentrations of 2′-FL during pregnancy tended to be consistently negatively associated with insulin levels (39).

The strong association between IR and NAFLD development has been linked to the involvement of bioactive lipids like DAGs in the etiology of the disease. DAGs can directly impair the insulin receptor (INSR) activity *via* recruitment of Protein kinase C delta (PRKCD) (3). Protein kinase C members are enzymes that are able to interact with lipids and affect cellular signaling cascades including insulin receptor signaling through kinase activity, a mechanism which occurs in various organs including liver and adipose tissue. PRKCD interacts with membrane-bound DAGs and blocks insulin signaling at the level of its receptor thereby promoting IR development (32). Under conditions of IR glucose uptake from the circulation is impaired and so is the synthesis of glycogen (1). In a state of hepatic IR, in which insulin-mediated activation of glycogen synthase is impaired, dietary glucose moieties will be redirected into the *de novo* lipogenesis pathway thereby further promoting lipid accumulation in NAFLD (1). The reduction of hyperinsulinemia and associated hepatic SREBF1 activity observed herein, indicate that hepatic insulin signaling may be improved with 2′-FL, supported by the significant reduction of hepatic DAG. Separate studies with dedicated sacrifice conditions are required to investigate putative effects on insulin signaling in more detail.

Another potential beneficial effect of 2′-FL that may have contributed to the observed decrease in steatosis is the counter regulation of several important factors associated with ER stress such as ATF4, ATF6, ERN1, NUPR1 indicating an overall attenuation of the ER stress response. The endoplasmic reticulum (ER) is an organelle that synthesizes, folds and secretes various proteins, among which are proteins to enable secretion of hepatic lipids. Thus, HFD-evoked ER stress can result in impaired protein folding and function, which can diminish lipid mobilization and thereby exacerbating the accumulation of intrahepatic lipids (40). Interestingly, one of the primary sites of DAG synthesis is in the ER (41) and accumulated DAGs in the ER were identified as key signaling molecules that cause disruption of the endomembrane system of the ER ultimately leading to induction of aforementioned factors implicated in ER stress (42), which is consistent with the HFD effects herein. It is thus amenable that the observed intrahepatic DAG accumulation upon HFD feeding is tightly associated with the induction of an ER stress response. 2′-FL lowered intrahepatic DAGs which may explain the counter-regulatory effect on the upstream regulators involved in the ER-stress response. This effect together with the improvement in lipid handling (i.e., activation of ACOX1 and deactivation of SREBF1) would reduce the export of lipids from the liver (as VLDL) and could therefore explain the slightly reduced triglyceride content in VLDL particles observed in the lipoprotein profile analysis. The ER stress-inducing effects of HFD may contribute to mitochondrial dysfunction also in another way because proper mitochondrial functioning (e.g., the Krebs cycle) relies on mitochondrial uptake of calcium from the ER (42, 43) which is disturbed under conditions of metabolic stress (44). Taken together, the combination of functional transcriptomics analysis with lipidomics for quantification of DAG concentrations in the same tissue provides a mechanistic rationale for the improvements in steatosis with 2′-FL and supports our histological observations in liver.

Besides the effects of 2′-FL on the liver we also showed that this prebiotic attenuated HFD-induced gut dysbiosis, in particular at the early time point (week 12). A pronounced microbiota dissimilarity was observed at week 16 and this was associated with a transient improvement of *in vivo* gut permeability during NAFLD development. In line with observations made in obese children treated with 2′-FL for 8-weeks, which resulted in increased intestinal abundance of Bifidobacterium (45), we also observed a gradual increase in Bifidobacterium over time. This is noteworthy, because 2′-FL seems to create a condition that facilitates persistent growth of Bifidobacterium while the total microbiome composition shifted back in the direction of HFD controls, possibly due to the very long period of experimental HFD feeding of more than 6 months and the associated severe metabolic stress. The observed effects on gut permeability are consistent with other rodent studies, which reported that preventive 2′-FL treatment for 6–8 weeks in HFD fed animals led to changes in the gut microbiota composition (33) and gut barrier function using *ex vivo* gut transplants (34). Microbiota dysbiosis and disturbed gut permeability are thought to be drivers of NASH pathology and reportedly precede the pathological manifestation of NAFLD (13, 14, 46). Gut permeability has even been associated with the severity of NAFLD (47, 48). The observed beneficial effects on the gut (permeability, microbiota shift and increased Bifidobacterium) with 2′-FL may therefore have contributed to the attenuation of NAFLD.

A limitation of the current study is that we did not sacrifice a group of mice at 8 weeks, the time point at which interventions were started which would have enabled us to assess NAFLD development prior to treatment to determine potential regression of histological features. Intrinsic to the set-up of the study, using 1oral administration of 2′-FL, we cannot distinguish whether the effects on liver or gut are direct, e.g., *via* 2′-FL itself or metabolites formed from 2′-FL, or more indirect, i.e., mediated *via* other metabolites that do not directly stem from 2′-FL but are produced by the altered microbiota as a whole. Consequently, the study design does also not allow investigation of the exact molecular mechanisms that mediate gut permeability effects, but this has been studied by others: 2′-FL directly increased expression of tight junction expression proteins, involved in the regulation of gut barrier integrity, in LPS-treated Caco2 cells (49) and directly diminished LPS-induced inflammation in human enterocytes (50) and Caco2 cells (49). Indirect effects of 2′-FL have been demonstrated by using 2′-FL derived bacterial metabolites, which attenuated paracellular permeability of FITC-dextran’s in Caco2 monolayers and upregulated the expression of tight junction proteins in a gut-on-a-chip model generated from human gut tissue biopsies (51). Taken together, both direct and indirect effects of 2′-FL on the gut may have contributed to the observed beneficial effects of 2′-FL.

In conclusion, we provide evidence that 2′-FL can exert effects beyond the gut and can affect more distant organs like the liver in which it improved lipid handling and reduced obesity-associated steatosis. The observed reduction of specific bioactive lipids in the liver, DAGs, constitutes an important finding because this class of lipids affects insulin signaling, is implicated in ER stress and contributes to the dysmetabolic state that causes insulin resistance. Hyperinsulinemia correlated with intrahepatic DAGs, and 2′-FL treatment reduced DAGs as well as HOMA-IR and mediators of ER stress. The improvement in microvesicular steatosis with 2′-FL is supported by improved lipid catabolism and suppression of *de novo* lipogenesis signaling pathways. Thus specific HMOs such as 2′-FL may constitute a means to correct gut dysfunction, dysmetabolism and insulin resistance, all of which drive the development of metabolic diseases including NAFLD.

## Data Availability Statement

The datasets presented in this study can be found in online repositories. The names of the repository/repositories and accession number(s) can be found in the article/Supplementary Material.

## Ethics Statement

The animal experiment was performed in accordance with the rules and regulations set forward by the Netherlands Law on Animal Experiments with ethical approval by an Independent Animal Welfare Body (IVD TNO; approval number 3682/TNO-269).

## Author Contributions

KS, RK, YQ, MM, and IB-P contributed to the conception and design of the study. JA, CR, EG, MG, and FS performed the research and acquired the data. KS, MC, and EG performed the statistical analysis. RK, EG, KS, YQ, and MH contributed to the analyses and interpretation of the data. RK, EG, and KS wrote the first draft of the manuscript. YQ and MH were involved in the study design, data analysis and decision to publish. All authors contributed to the finalization of the manuscript, and have read and approved the submitted version.

## Conflict of Interest

YQ has been a full-time employee at BASF South East Asia Pte Ltd., Singapore. MH has been a full-time employee at BASF SE, Germany. The remaining authors declare that the research was conducted in the absence of any commercial or financial relationships that could be construed as a potential conflict of interest. The authors declare that this study received funding from BASF as one of the partners of the public-private partnership (PPP) ProLiver. The funder BASF was involved in the study design, data analysis and interpretation, decision to publish and preparation of the manuscript.

## Publisher’s Note

All claims expressed in this article are solely those of the authors and do not necessarily represent those of their affiliated organizations, or those of the publisher, the editors and the reviewers. Any product that may be evaluated in this article, or claim that may be made by its manufacturer, is not guaranteed or endorsed by the publisher.
