# Supplementary material for: The Human Milk Oligosaccharide 2′-Fucosyllactose Alleviates Liver Steatosis, ER Stress and Insulin Resistance by Reducing Hepatic Diacylglycerols and Improved Gut Permeability in Obese Ldlr-/-.Leiden Mice
Source: Front Nutr. 2022 Jun 17;9:904740. doi: 10.3389/fnut.2022.904740 (PMC9248376; doi:10.3389/fnut.2022.904740)
Supplement: Supplementary file 1 [file Table_1.DOCX]

Supplementary Material

**Table 1**. Canonical pathway analysis in the liver

|  | **HFD vs Chow** | | **HFD + 2’-FL vs HFD** | |
| --- | --- | --- | --- | --- |
| **Canonical Pathways** | **Z-score** | **p-value** | **Z-score** | **p-value** |
| NRF2-mediated Oxidative Stress Response | 1.4 | 0.000 | -1.6 | 0.000 |
| Thrombin Signaling | 2.3 | 0.000 | -1.0 | 0.241 |
| Cardiac Hypertrophy Signaling | 1.9 | 0.024 | -1.0 | 0.326 |
| Glioblastoma Multiforme Signaling | 2.2 | 0.000 | 0.0 | 0.015 |
| B Cell Receptor Signaling | 2.2 | 0.000 | 0.0 | 0.073 |
| CXCR4 Signaling | 2.2 | 0.004 | 0.0 | 0.142 |
| Endothelin-1 Signaling | 2.1 | 0.001 | 0.0 | 0.190 |
| ILK Signaling | 3.2 | 0.000 | 0.0 | 0.195 |
| Protein Kinase A Signaling | 0.4 | 0.014 | 0.0 | 0.204 |
| Adrenomedullin signaling pathway | 2.9 | 0.001 | 0.0 | 0.213 |
| Role of NFAT in Cardiac Hypertrophy | 2.2 | 0.027 | 0.0 | 0.256 |
| Opioid Signaling Pathway | 1.3 | 0.012 | 0.0 | 0.345 |
| Colorectal Cancer Metastasis Signaling | 4.4 | 0.000 | 0.0 | 0.361 |
| Systemic Lupus Erythematosus In B Cell Signaling Pathway | 4.1 | 0.000 | 0.0 | 0.421 |
| Cardiac Hypertrophy Signaling (Enhanced) | 3.2 | 0.009 | 0.0 | 0.532 |
| Hepatic Fibrosis Signaling Pathway | 4.4 | 0.000 | 0.0 | 1.000 |
| Sirtuin Signaling Pathway | 0.5 | 0.000 | 0.4 | 0.025 |
| Senescence Pathway | 1.9 | 0.000 | 0.8 | 0.115 |
| HOTAIR Regulatory Pathway | 1.5 | 0.016 | 1.0 | 0.043 |
| FGF Signaling | -0.3 | 0.353 | 1.3 | 0.003 |
| PXR/RXR Activation | N/A | 0.001 | N/A | 0.001 |
| Endoplasmic Reticulum Stress Pathway | -0.7 | 0.012 | N/A | 0.002 |
| UDP-N-acetyl-D-galactosamine Biosynthesis II | N/A | 1.000 | N/A | 0.005 |
| Estrogen Receptor Signaling | N/A | 0.004 | N/A | 0.006 |
| Remodeling of Epithelial Adherens Junctions | 1.1 | 0.112 | N/A | 0.009 |
| tRNA Charging | -1.4 | 0.272 | N/A | 0.011 |
| UDP-N-acetyl-D-galactosamine Biosynthesis I | N/A | 1.000 | N/A | 0.012 |
| Hypoxia Signaling in the Cardiovascular System | 1.4 | 0.000 | N/A | 0.012 |
| IL-7 Signaling Pathway | 0.7 | 0.034 | N/A | 0.014 |
| Renal Cell Carcinoma Signaling | 1.8 | 0.006 | N/A | 0.016 |
| PFKFB4 Signaling Pathway | 1.7 | 0.302 | N/A | 0.018 |
| 14-3-3-mediated Signaling | 2.0 | 0.001 | N/A | 0.019 |
| Xenobiotic Metabolism Signaling | N/A | 0.000 | N/A | 0.023 |
| Regulation of the Epithelial-Mesenchymal Transition Pathway | N/A | 1.000 | N/A | 0.029 |
| Aryl Hydrocarbon Receptor Signaling | 0.2 | 0.000 | N/A | 0.029 |
| Unfolded protein response | -1.1 | 0.012 | N/A | 0.030 |
| UVA-Induced MAPK Signaling | 2.1 | 0.000 | N/A | 0.031 |
| Gap Junction Signaling | N/A | 0.000 | N/A | 0.033 |
| Glutathione Redox Reactions I | 0.0 | 0.170 | N/A | 0.033 |
| Breast Cancer Regulation by Stathmin1 | N/A | 0.000 | N/A | 0.034 |
| Glutathione Biosynthesis | N/A | 0.068 | N/A | 0.036 |
| N-acetylglucosamine Degradation I | N/A | 0.405 | N/A | 0.036 |
| 1,25-dihydroxyvitamin D3 Biosynthesis | N/A | 0.405 | N/A | 0.036 |
| Sumoylation Pathway | -2.1 | 0.002 | N/A | 0.036 |
| Epithelial Adherens Junction Signaling | N/A | 0.035 | N/A | 0.037 |
| Lipid Antigen Presentation by CD1 | N/A | 0.106 | N/A | 0.039 |
| Aldosterone Signaling in Epithelial Cells | 0.6 | 0.000 | N/A | 0.042 |
| eNOS Signaling | 2.4 | 0.089 | N/A | 0.043 |
| Pyridoxal 5'-phosphate Salvage Pathway | 0.7 | 0.001 | N/A | 0.043 |
| N-acetylglucosamine Degradation II | N/A | 0.121 | N/A | 0.047 |
| Glutathione Redox Reactions II | N/A | 0.499 | N/A | 0.047 |
| Protein Ubiquitination Pathway | N/A | 0.000 | N/A | 0.047 |

*The activity of a canonical pathways was predicted based on gene expression changes of downstream target genes. The provided list here is a selection of pathways in which the pathway had either a Z-score or P-value with 2’-FL. A negative Z-score indicates inhibition of the respective pathway and a positive Z-score indicates activation. The p-value < 0.05 indicates significant enrichment of the target genes downstream, i.e. that more downstream genes are affected than can be expected by chance. N/A indicates an insufficient number of differentially expressed genes to predict the activation state of a pathway*.
